# Crystal structure of *N* ^1^-phenyl-*N* ^4^-[(quinolin-2-yl)methyl­idene]benzene-1,4-di­amine

**DOI:** 10.1107/S1600536814016006

**Published:** 2014-08-01

**Authors:** Md. Serajul Haque Faizi, Ashraf Mashrai, Saleem Garandal, M. Shahid

**Affiliations:** a Department of Chemistry, Indian Institute of Technology Kanpur, Kanpur, UP 208 016, India; b Department of Chemistry, Aligarh Muslim University, Aligarh 202 002, India; c School of Chemical Sciences, S.R.T.M. University, Nanded 431 606, India

**Keywords:** crystal structure, quinoline, C—H⋯π inter­actions

## Abstract

In the title compound, C_22_H_17_N_3_, the dihedral angles between the central benzene ring and the terminal phenyl ring and quinoline ring system (r.m.s. deviation = 0.027 Å) are 44.72 (7) and 9.02 (4)°, respectively, and the bond-angle sum at the amine N atom is 359.9°. In the crystal, the N—H group is not involved in hydrogen bonding and the mol­ecules are linked by weak C—H⋯π inter­actions, generating [010] chains.

## Related literature   

For applications of quinoline-containing Schiff bases see: Das *et al.* (2013 ▶); Jursic *et al.* (2002 ▶); Motswainyana *et al.* (2013 ▶); Song *et al.* (2011 ▶). The present work is part of an ongoing structural study of Schiff base-metal complexes, see: Faizi & Hussain (2014 ▶); Faizi & Sen (2014 ▶); Faizi *et al.* (2014 ▶).
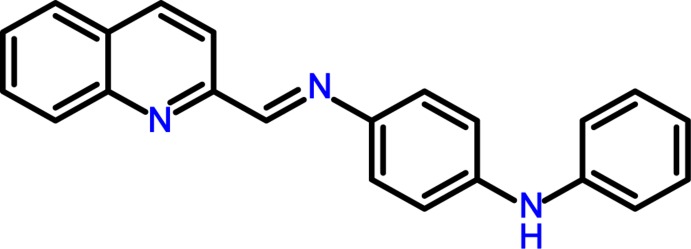


## Experimental   

### Crystal data  

C_22_H_17_N_3_
*M* *_r_* = 323.39
Monoclinic, 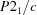

*a* = 17.595 (2) Å
*b* = 7.3348 (8) Å
*c* = 12.5712 (18) Å
β = 99.769 (6)°
*V* = 1598.9 (4) Å^3^
*Z* = 4
Mo *K*α radiation
μ = 0.08 mm^−1^
*T* = 100 K
0.29 × 0.21 × 0.15 mm

### Data collection  

Bruker SMART APEX CCD diffractometer
Absorption correction: multi-scan (*SADABS*; Sheldrick, 2004 ▶) *T*_min_ = 0.967, *T*_max_ = 0.984
6866 measured reflections
2964 independent reflections
1557 reflections with *I* > 2σ(*I*)
*R*_int_ = 0.063

### Refinement  

*R*[*F* ^2^ > 2σ(*F* ^2^)] = 0.054
*wR*(*F* ^2^) = 0.105
*S* = 0.97
2964 reflections
234 parameters
H atoms treated by a mixture of independent and constrained refinement
Δρ_max_ = 0.22 e Å^−3^
Δρ_min_ = −0.19 e Å^−3^

### 

Data collection: *SMART* (Bruker, 2003 ▶); cell refinement: *SAINT* (Bruker, 2003 ▶); data reduction: *SAINT*; program(s) used to solve structure: *SIR97* (Altomare *et al.*, 1999 ▶); program(s) used to refine structure: *SHELXL97* (Sheldrick, 2008 ▶); molecular graphics: *DIAMOND* (Brandenberg & Putz, 2006 ▶); software used to prepare material for publication: *DIAMOND*.

## Supplementary Material

Crystal structure: contains datablock(s) global, I. DOI: 10.1107/S1600536814016006/hb7248sup1.cif

Structure factors: contains datablock(s) I. DOI: 10.1107/S1600536814016006/hb7248Isup2.hkl

Supporting information file. DOI: 10.1107/S1600536814016006/hb7248Isup3.cml

**Fig. 1.**
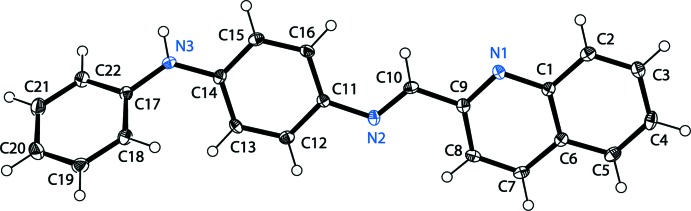
The molecular structure of the title compound, with non-H atoms drawn as 40% probability displacement ellipsoids.

. DOI: 10.1107/S1600536814016006/hb7248fig1.tif
The mol­ecular structure of the title compound, with non-H atoms drawn as 40% probability displacement ellipsoids.

CCDC reference: 1012864

Additional supporting information:  crystallographic information; 3D view; checkCIF report

## Figures and Tables

**Table 1:** Hydrogen-bond geometry (Å, °) *Cg*1, *Cg*2 and *Cg*3 are the centroids of the N1/C1/C6–C9, C1–C6 and C11–C16 rings, respectively.

*D*—H⋯*A*	*D*—H	H⋯*A*	*D*⋯*A*	*D*—H⋯*A*
C7—H7⋯*Cg*3^i^	0.93	2.61	3.430 (2)	148
C12—H12⋯*Cg*1^i^	0.93	2.79	3.536 (2)	138
C13—H13⋯*Cg*2^i^	0.93	2.71	3.508 (3)	145

Symmetry code: (i) 
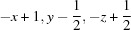
.

## supplementary crystallographic information

### S1. Chemical context

Quinoline derivatives of Schiff bases are important building blocks of many important compounds widely used in biological applications such as anti­oxidative and anti­cancer and fluorescent probe agents in industry and in coordination chemistry (Motswainyana *et al.*, 2013; Das *et al.*, 2013; Song *et al.*, 2011; Jursic *et al.*, 2002). The present work is part of an ongoing structural study of Schiff base metal complexes (Faizi & Hussain, 2014; Faizi & Sen, 2014; Faizi *et al.* 2014) and we report here the structure of *N*^1^-phenyl-*N*^4^-[(quinolin-2-yl)methyl­idene]benzene-1,4-di­amine (PQMBD).

### S2. Structural commentary

The synthesis of PQMBD by condensation of 2-quinoline­carboxaldehyde and *N*-phenyl-*p*-phenyl­enedi­amine has not previously been reported. In the title compound (Fig. 1) PQMBD has non planar structure, the dihedral angle between the quinolinyl and *p*-phenyl­enedi­amine rings is 9.02 (4)° and the dihedral angle between the *p*-phenyl­enedi­amine and *N*-phenyl rings is 44.72 (7)°. The imine group displays a torsional angle (C9—C10—N2—C11) of 179.20 (2)°.

### S3. Supra­molecular features

In the crystal, the N—H group is not involved in hydrogen bonding and the molecules are linked by weak C—H···π inter­actions, generating [010] chains.

### S4. Database survey

There are very few examples similar to title compound and their metal complex have been reported in the literature (Patra & Goldberg 2003; Gonzalez *et al.*, 2012).

### S5. Synthesis and crystallization

100 mg (1 mmol) of *N*-phenyl-*p*-phenyl­enedi­amine were dissolved in 10 ml of absolute ethanol. To this solution, 85 mg (1 mmol) of 2-quinoline­carboxaldehyde in 5 ml of absolute ethanol was dropwisely added under stirring. Then, this mixture was stirred for 10 min, two drops of glacial acetic acid were then added and the mixture was further refluxed for 2 h. The resulting yellow precipitate was recovered by filtration, washed several times with a small portions of EtOH and then with di­ethyl ether to give 150 mg (88%) of the title compound. Yellow blocks were obtained within 3 days by slow evaporation of the MeOH solvent.

### S6. Refinement

Crystal data, data collection and structure refinement details are summarized in Table 1. the N-bound H-atoms were located in difference Fourier maps,and their positions were then held fixed. All H-atoms were positioned geometrically and refined using a riding model with C—H = 0.92–0.93 Å and *U*_iso_(H) = 1.2*U*_eq_(C).

### Crystal data

**Table:**

C_22_H_17_N_3_	*F*(000) = 680
*M**_r_* = 323.39	*D*_x_ = 1.343 Mg m^−^^3^
Monoclinic, *P*2_1_/*c*	Mo *K*α radiation, λ = 0.71073 Å
Hall symbol: -P 2ybc	Cell parameters from 999 reflections
*a* = 17.595 (2) Å	θ = 2.6–28.6°
*b* = 7.3348 (8) Å	µ = 0.08 mm^−^^1^
*c* = 12.5712 (18) Å	*T* = 100 K
β = 99.769 (6)°	Block, yellow
*V* = 1598.9 (4) Å^3^	0.29 × 0.21 × 0.15 mm
*Z* = 4	

### Data collection

**Table:**

Bruker SMART APEX CCD diffractometer	2964 independent reflections
Radiation source: fine-focus sealed tube	1557 reflections with *I* > 2σ(*I*)
Graphite monochromator	*R*_int_ = 0.063
ω–scans	θ_max_ = 25.5°, θ_min_ = 2.4°
Absorption correction: multi-scan (*SADABS*; Sheldrick, 2004)	*h* = −19→21
*T*_min_ = 0.967, *T*_max_ = 0.984	*k* = −8→8
6866 measured reflections	*l* = −12→15

### Refinement

**Table:**

Refinement on *F*^2^	Primary atom site location: structure-invariant direct methods
Least-squares matrix: full	Secondary atom site location: difference Fourier map
*R*[*F*^2^ > 2σ(*F*^2^)] = 0.054	Hydrogen site location: inferred from neighbouring sites
*wR*(*F*^2^) = 0.105	H atoms treated by a mixture of independent and constrained refinement
*S* = 0.97	*w* = 1/[σ^2^(*F*_o_^2^) + (0.033*P*)^2^] where *P* = (*F*_o_^2^ + 2*F*_c_^2^)/3
2964 reflections	(Δ/σ)_max_ < 0.001
234 parameters	Δρ_max_ = 0.22 e Å^−^^3^
0 restraints	Δρ_min_ = −0.19 e Å^−^^3^

### Special details

**Table:**

Geometry. All e.s.d.'s (except the e.s.d. in the dihedral angle between two l.s. planes) are estimated using the full covariance matrix. The cell e.s.d.'s are taken into account individually in the estimation of e.s.d.'s in distances, angles and torsion angles; correlations between e.s.d.'s in cell parameters are only used when they are defined by crystal symmetry. An approximate (isotropic) treatment of cell e.s.d.'s is used for estimating e.s.d.'s involving l.s. planes.
Refinement. Refinement of *F*^2^ against ALL reflections. The weighted *R*-factor *wR* and goodness of fit *S* are based on *F*^2^, conventional *R*-factors *R* are based on *F*, with *F* set to zero for negative *F*^2^. The threshold expression of *F*^2^ > σ(*F*^2^) is used only for calculating *R*-factors(gt) *etc*. and is not relevant to the choice of reflections for refinement. *R*-factors based on *F*^2^ are statistically about twice as large as those based on *F*, and *R*- factors based on ALL data will be even larger.

### Fractional atomic coordinates and isotropic or equivalent isotropic displacement parameters (Å^2^)

**Table:**

	*x*	*y*	*z*	*U*_iso_*/*U*_eq_	
C1	0.25415 (13)	0.2764 (3)	0.16305 (19)	0.0148 (6)	
C2	0.18560 (13)	0.3785 (3)	0.1579 (2)	0.0186 (6)	
H2	0.1771	0.4464	0.2173	0.022*	
C3	0.13189 (14)	0.3776 (3)	0.0660 (2)	0.0209 (6)	
H3	0.0869	0.4454	0.0635	0.025*	
C4	0.14301 (14)	0.2766 (3)	−0.0251 (2)	0.0215 (7)	
H4	0.1059	0.2777	−0.0873	0.026*	
C5	0.20884 (14)	0.1767 (3)	−0.0211 (2)	0.0208 (6)	
H5	0.2164	0.1100	−0.0813	0.025*	
C6	0.26537 (14)	0.1730 (3)	0.07237 (19)	0.0155 (6)	
C7	0.33399 (13)	0.0704 (3)	0.08109 (19)	0.0173 (6)	
H7	0.3429	−0.0027	0.0240	0.021*	
C8	0.38676 (14)	0.0787 (3)	0.1728 (2)	0.0174 (6)	
H8	0.4318	0.0101	0.1798	0.021*	
C9	0.37258 (14)	0.1929 (3)	0.2577 (2)	0.0157 (6)	
C10	0.43043 (15)	0.2137 (3)	0.3554 (2)	0.0163 (6)	
C11	0.55486 (14)	0.1692 (3)	0.4537 (2)	0.0138 (6)	
C12	0.62578 (13)	0.0883 (3)	0.44931 (19)	0.0179 (6)	
H12	0.6325	0.0252	0.3874	0.022*	
C13	0.68652 (13)	0.0993 (3)	0.5345 (2)	0.0182 (6)	
H13	0.7332	0.0432	0.5296	0.022*	
C14	0.67803 (14)	0.1942 (3)	0.62763 (19)	0.0159 (6)	
C15	0.60659 (14)	0.2723 (3)	0.6334 (2)	0.0182 (6)	
H15	0.5996	0.3345	0.6955	0.022*	
C16	0.54638 (13)	0.2585 (3)	0.54833 (19)	0.0180 (6)	
H16	0.4990	0.3101	0.5543	0.022*	
C17	0.81614 (14)	0.1848 (3)	0.7206 (2)	0.0159 (6)	
C18	0.85238 (14)	0.2256 (3)	0.6337 (2)	0.0196 (6)	
H18	0.8234	0.2653	0.5691	0.023*	
C19	0.93117 (15)	0.2075 (3)	0.6429 (2)	0.0246 (7)	
H19	0.9548	0.2354	0.5841	0.030*	
C20	0.97548 (15)	0.1486 (3)	0.7378 (2)	0.0286 (7)	
H20	1.0285	0.1349	0.7431	0.034*	
C21	0.93964 (15)	0.1103 (3)	0.8252 (2)	0.0282 (7)	
H21	0.9690	0.0731	0.8901	0.034*	
C22	0.86074 (14)	0.1270 (3)	0.8167 (2)	0.0211 (7)	
H22	0.8372	0.0993	0.8756	0.025*	
N1	0.30802 (11)	0.2868 (2)	0.25571 (15)	0.0160 (5)	
N2	0.49805 (11)	0.1482 (2)	0.36031 (15)	0.0165 (5)	
N3	0.73653 (12)	0.2067 (3)	0.71688 (18)	0.0206 (6)	
H3N	0.7212 (13)	0.233 (3)	0.779 (2)	0.034 (8)*	
H10	0.4135 (11)	0.284 (3)	0.4156 (16)	0.016 (6)*	

### Atomic displacement parameters (Å^2^)

**Table:**

	*U*^11^	*U*^22^	*U*^33^	*U*^12^	*U*^13^	*U*^23^
C1	0.0146 (16)	0.0161 (14)	0.0142 (15)	−0.0034 (12)	0.0040 (12)	0.0024 (11)
C2	0.0214 (16)	0.0176 (14)	0.0176 (17)	−0.0046 (12)	0.0052 (13)	0.0003 (12)
C3	0.0174 (16)	0.0222 (15)	0.0226 (18)	0.0018 (12)	0.0021 (13)	0.0023 (13)
C4	0.0190 (17)	0.0225 (15)	0.0204 (17)	−0.0068 (13)	−0.0038 (12)	0.0032 (13)
C5	0.0271 (17)	0.0182 (15)	0.0172 (17)	−0.0056 (13)	0.0041 (13)	−0.0017 (12)
C6	0.0168 (16)	0.0146 (14)	0.0154 (16)	−0.0047 (11)	0.0036 (12)	0.0020 (12)
C7	0.0236 (16)	0.0152 (14)	0.0141 (16)	−0.0031 (12)	0.0063 (13)	−0.0006 (11)
C8	0.0189 (16)	0.0151 (14)	0.0200 (17)	0.0023 (11)	0.0086 (13)	0.0019 (12)
C9	0.0157 (16)	0.0138 (14)	0.0183 (16)	−0.0033 (12)	0.0045 (12)	0.0027 (12)
C10	0.0195 (17)	0.0123 (14)	0.0180 (17)	−0.0002 (12)	0.0056 (13)	−0.0014 (12)
C11	0.0132 (15)	0.0132 (14)	0.0159 (16)	−0.0024 (11)	0.0046 (12)	0.0022 (11)
C12	0.0227 (16)	0.0133 (14)	0.0182 (17)	0.0010 (12)	0.0045 (13)	−0.0013 (11)
C13	0.0141 (16)	0.0167 (14)	0.0236 (18)	0.0053 (11)	0.0027 (13)	0.0018 (12)
C14	0.0180 (16)	0.0155 (14)	0.0134 (16)	−0.0029 (12)	0.0007 (12)	0.0041 (12)
C15	0.0234 (17)	0.0144 (14)	0.0176 (16)	−0.0012 (12)	0.0058 (13)	−0.0013 (11)
C16	0.0160 (16)	0.0205 (15)	0.0190 (16)	−0.0006 (11)	0.0069 (13)	−0.0006 (12)
C17	0.0158 (16)	0.0132 (14)	0.0181 (16)	−0.0023 (11)	0.0007 (12)	−0.0015 (12)
C18	0.0189 (17)	0.0185 (14)	0.0206 (17)	−0.0019 (12)	0.0012 (13)	0.0001 (12)
C19	0.0248 (17)	0.0296 (16)	0.0206 (17)	−0.0071 (13)	0.0070 (13)	−0.0038 (13)
C20	0.0177 (17)	0.0350 (17)	0.033 (2)	−0.0028 (13)	0.0045 (15)	−0.0060 (14)
C21	0.0247 (18)	0.0319 (17)	0.0254 (19)	0.0011 (13)	−0.0031 (14)	−0.0006 (13)
C22	0.0209 (17)	0.0212 (15)	0.0210 (18)	−0.0025 (12)	0.0030 (13)	0.0022 (12)
N1	0.0139 (13)	0.0151 (11)	0.0190 (14)	−0.0002 (10)	0.0026 (10)	0.0026 (9)
N2	0.0152 (13)	0.0157 (12)	0.0179 (14)	−0.0014 (9)	0.0005 (10)	0.0025 (9)
N3	0.0185 (14)	0.0302 (14)	0.0137 (14)	0.0002 (10)	0.0041 (11)	−0.0032 (11)

### Geometric parameters (Å, º)

**Table:**

C1—N1	1.373 (3)	C12—C13	1.381 (3)
C1—C6	1.411 (3)	C12—H12	0.9300
C1—C2	1.412 (3)	C13—C14	1.392 (3)
C2—C3	1.363 (3)	C13—H13	0.9300
C2—H2	0.9300	C14—N3	1.391 (3)
C3—C4	1.405 (3)	C14—C15	1.394 (3)
C3—H3	0.9300	C15—C16	1.375 (3)
C4—C5	1.364 (3)	C15—H15	0.9300
C4—H4	0.9300	C16—H16	0.9300
C5—C6	1.405 (3)	C17—C18	1.387 (3)
C5—H5	0.9300	C17—C22	1.391 (3)
C6—C7	1.411 (3)	C17—N3	1.403 (3)
C7—C8	1.354 (3)	C18—C19	1.378 (3)
C7—H7	0.9300	C18—H18	0.9300
C8—C9	1.412 (3)	C19—C20	1.380 (3)
C8—H8	0.9300	C19—H19	0.9300
C9—N1	1.325 (3)	C20—C21	1.384 (3)
C9—C10	1.464 (3)	C20—H20	0.9300
C10—N2	1.275 (3)	C21—C22	1.380 (3)
C10—H10	1.00 (2)	C21—H21	0.9300
C11—C16	1.388 (3)	C22—H22	0.9300
C11—C12	1.391 (3)	N3—H3N	0.89 (2)
C11—N2	1.415 (3)		
			
N1—C1—C6	123.0 (2)	C12—C13—C14	120.1 (2)
N1—C1—C2	118.1 (2)	C12—C13—H13	119.9
C6—C1—C2	118.9 (2)	C14—C13—H13	119.9
C3—C2—C1	120.0 (2)	N3—C14—C13	122.8 (2)
C3—C2—H2	120.0	N3—C14—C15	118.8 (2)
C1—C2—H2	120.0	C13—C14—C15	118.4 (2)
C2—C3—C4	121.4 (2)	C16—C15—C14	120.8 (2)
C2—C3—H3	119.3	C16—C15—H15	119.6
C4—C3—H3	119.3	C14—C15—H15	119.6
C5—C4—C3	119.2 (2)	C15—C16—C11	121.4 (2)
C5—C4—H4	120.4	C15—C16—H16	119.3
C3—C4—H4	120.4	C11—C16—H16	119.3
C4—C5—C6	121.1 (2)	C18—C17—C22	118.9 (2)
C4—C5—H5	119.4	C18—C17—N3	122.6 (2)
C6—C5—H5	119.4	C22—C17—N3	118.5 (2)
C5—C6—C1	119.3 (2)	C19—C18—C17	120.3 (2)
C5—C6—C7	123.4 (2)	C19—C18—H18	119.9
C1—C6—C7	117.3 (2)	C17—C18—H18	119.9
C8—C7—C6	119.7 (2)	C18—C19—C20	121.0 (3)
C8—C7—H7	120.1	C18—C19—H19	119.5
C6—C7—H7	120.1	C20—C19—H19	119.5
C7—C8—C9	119.2 (2)	C19—C20—C21	118.9 (3)
C7—C8—H8	120.4	C19—C20—H20	120.6
C9—C8—H8	120.4	C21—C20—H20	120.6
N1—C9—C8	123.7 (2)	C22—C21—C20	120.6 (3)
N1—C9—C10	115.7 (2)	C22—C21—H21	119.7
C8—C9—C10	120.6 (2)	C20—C21—H21	119.7
N2—C10—C9	120.8 (2)	C21—C22—C17	120.4 (3)
N2—C10—H10	123.5 (12)	C21—C22—H22	119.8
C9—C10—H10	115.7 (12)	C17—C22—H22	119.8
C16—C11—C12	117.6 (2)	C9—N1—C1	117.0 (2)
C16—C11—N2	126.8 (2)	C10—N2—C11	121.4 (2)
C12—C11—N2	115.6 (2)	C14—N3—C17	128.1 (2)
C13—C12—C11	121.7 (2)	C14—N3—H3N	115.3 (16)
C13—C12—H12	119.1	C17—N3—H3N	116.5 (16)
C11—C12—H12	119.1		
			
N1—C1—C2—C3	177.5 (2)	C13—C14—C15—C16	−1.1 (3)
C6—C1—C2—C3	−0.7 (3)	C14—C15—C16—C11	−1.0 (3)
C1—C2—C3—C4	0.0 (4)	C12—C11—C16—C15	2.2 (3)
C2—C3—C4—C5	0.3 (4)	N2—C11—C16—C15	−179.0 (2)
C3—C4—C5—C6	0.2 (4)	C22—C17—C18—C19	0.5 (3)
C4—C5—C6—C1	−0.9 (3)	N3—C17—C18—C19	177.8 (2)
C4—C5—C6—C7	179.2 (2)	C17—C18—C19—C20	0.1 (4)
N1—C1—C6—C5	−177.0 (2)	C18—C19—C20—C21	−1.0 (4)
C2—C1—C6—C5	1.1 (3)	C19—C20—C21—C22	1.4 (4)
N1—C1—C6—C7	2.9 (3)	C20—C21—C22—C17	−0.8 (4)
C2—C1—C6—C7	−179.0 (2)	C18—C17—C22—C21	−0.1 (3)
C5—C6—C7—C8	177.9 (2)	N3—C17—C22—C21	−177.5 (2)
C1—C6—C7—C8	−2.0 (3)	C8—C9—N1—C1	−2.9 (3)
C6—C7—C8—C9	−1.0 (3)	C10—C9—N1—C1	177.1 (2)
C7—C8—C9—N1	3.7 (3)	C6—C1—N1—C9	−0.5 (3)
C7—C8—C9—C10	−176.3 (2)	C2—C1—N1—C9	−178.6 (2)
N1—C9—C10—N2	−171.1 (2)	C9—C10—N2—C11	179.2 (2)
C8—C9—C10—N2	8.9 (3)	C16—C11—N2—C10	0.0 (3)
C16—C11—C12—C13	−1.5 (3)	C12—C11—N2—C10	178.8 (2)
N2—C11—C12—C13	179.6 (2)	C13—C14—N3—C17	22.2 (4)
C11—C12—C13—C14	−0.5 (3)	C15—C14—N3—C17	−161.0 (2)
C12—C13—C14—N3	178.6 (2)	C18—C17—N3—C14	29.4 (4)
C12—C13—C14—C15	1.8 (3)	C22—C17—N3—C14	−153.2 (2)
N3—C14—C15—C16	−178.0 (2)		

### Hydrogen-bond geometry (Å, º)

Cg1, Cg2 and Cg3 are the centroids of the N1/C1/C6–C9, C1–C6 and C11–C16 rings, respectively.

**Table:**

*D*—H···*A*	*D*—H	H···*A*	*D*···*A*	*D*—H···*A*
C7—H7···*Cg*3^i^	0.93	2.61	3.430 (2)	148
C12—H12···*Cg*1^i^	0.93	2.79	3.536 (2)	138
C13—H13···*Cg*2^i^	0.93	2.71	3.508 (3)	145

Symmetry code: (i) −*x*+1, *y*−1/2, −*z*+1/2.
