# Supplementary figures and images for: Crystal structure of N 1-phenyl-N 4-[(quinolin-2-yl)methyl­idene]benzene-1,4-di­amine
Source: Acta Crystallogr Sect E Struct Rep Online. 2014 Aug 1;70(Pt 9):o905–6. doi: 10.1107/S1600536814016006 (PMC4186095; doi:10.1107/S1600536814016006)

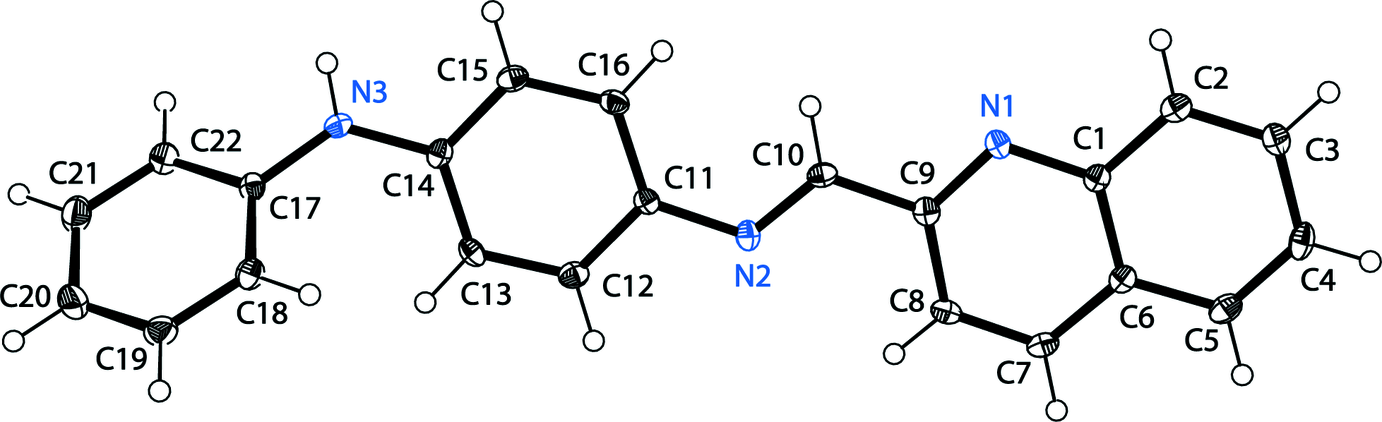

Supplement: Supplementary file 4 [file e-70-0o905-fig1.tif]
